# Differential distribution of IgA-protease genotypes in mucosal and invasive isolates of *Haemophilus influenzae* in Sweden

**DOI:** 10.1186/s12879-018-3464-3

**Published:** 2018-11-22

**Authors:** Fredrik Resman, Guillaume Manat, Victor Lindh, Timothy F. Murphy, Kristian Riesbeck

**Affiliations:** 1 Clinical Infection Medicine, Department of Translational Medicine, Lund University, Malmö, Sweden; 2 Riesbeck Lab, Clinical Microbiology, Department of Translational Medicine, Lund University, Jan Waldenströms gata 59, SE20502 Malmö, Sweden; 3 Clinical and Translational Research Center, University at Buffalo, the State University of New York, New York, USA

**Keywords:** Genotype, Human infection, *Haemophilus influenzae*, IgA protease

## Abstract

**Background:**

Several different IgA-proteases exist in *Haemophilus influenzae*. The variants have been suggested to play differential roles in pathogenesis, but there is limited information on their distribution in clinical isolates. The objective of this study was to investigate the distribution of IgA-protease genotypes in *H. influenzae* and assess the association between IgA-protease genotype and type of clinical infection.

**Methods:**

We performed PCR-screening of the IgA-protease gene variants in two cohorts of clinical *H. influenzae.* The first cohort consisted of 177 isolates from individuals with respiratory tract infection in January 2010, 2011 and 2012. Information on age, gender and clinical infection was available in this cohort. The second cohort comprised 53 isolates, including NTHi from bloodstream, cerebrospinal fluid (CSF) and urogenital origin as well as encapsulated isolates respresenting all capsule types. We assessed associations between IgA protease genotype and clinical predictors using basic statistical tests of association as well as regression analysis.

**Results:**

The *igaB* gene was found in 46% of isolates in the respiratory tract cohort, and no evident trend could be seen during the study years. However, the *igaB* gene was significantly less common among invasive isolates (19%), *p* = 0.003 (Fischer’s exact test), even when encapsulated isolates were excluded (21%), *p* = 0.012. A significantly negative association between bacteraemia and *igaB* genotype remained after adjusting for covariates. We did not identify a significant association between IgA-protease gene variants and type of respiratory tract infection, but isolates with an *igaA2* genotype were overrepresented in pre-school children.

**Conclusions:**

The distribution of IgA-protease gene variants in Swedish *H. influenzae* highlighted the widespread abundance of the *igaB* in isolates from cases of respiratory tract infection, but the *igaB* gene variant was significantly less common in invasive (bloodstream and CSF) isolates of *H. influenzae* compared with respiratory tract isolates.

**Electronic supplementary material:**

The online version of this article (10.1186/s12879-018-3464-3) contains supplementary material, which is available to authorized users.

## Background

A virulence factor common to most successful bacterial respiratory tract pathogens is the ability to degrade human IgA1, the dominant IgA subclass in the nasopharyngeal cavity [1]. Cleavage of IgA1 at different sites of the heavily glycosylated hinge region is performed by secreted endopeptidases that are structurally heterogenous in different pathogens. IgA-proteases are produced by *Streptococcus pneumoniae* [2], and presence of IgA proteases distinguish pathogenic from non-pathogenic species of *Neisseria* [3] and *Haemophili* [4]. The exact biological significance of microbial IgA-proteases is not understood, as animal models are of little use due to the specificity for human IgA1, but IgA-proteases are widely believed to facilitate host mucosal colonization and tissue invasion [5].

In *H. influenzae*, at least three patterns of IgA1 cleavage exist, defined by what bond they cleave in the hinge region; IgA can be cleaved at either of two distinct sites or at both sites simultaneously [6, 7]. Encapsulated *H. influenzae* are generally clonal and have specific cleavage patterns, even though data are conflicting regarding the cleavage pattern of type f isolates, the only encapsulated type associated with IgA-protease antigenic diversity [6–8]. In contrast, non-typeable *H. influenzae* isolates are heterogenous, may have any of the three cleavage patterns and have a high degree of IgA-protease antigenic diversity [7]. The cleavage patterns correspond to different gene variants of the common IgA-protease gene, *igaA*, and of a second IgA-protease gene, *igaB*, with a high degree of homology with the IgA1 protease of *Neisseria* spp. [9]. The two genes both code for autotransporters with autoproteolytic cleavage of the passenger domain responsible for the protease activity [10].

Virtually all *H. influenzae* isolates have the *iga* gene, while only a subgroup carries both *igaA* and *igaB*. The presence of *igaB* is associated with higher levels of protease activity and is considered to be a virulence factor on its own [11]. Altogether, four distinct gene variants of IgA-proteases have been discovered based on two variants of the two genes [12], and their respective expression results in at least three different cleavage patterns. In the only major study that has been performed regarding the IgA-protease gene distribution in a substantial collection of isolates, 30–40% of NTHi causing disease carried the *igaB* gene [11], with a distinct variation between disease entities.

The purposes of this study were: 1) to investigate the distribution of IgA-protease genotypes in a collection of consecutive clinical respiratory tract *H. influenzae* isolates from patients with respiratory tract infections as well as in a more diverse collection of encapsulated and invasive isolates, and 2) to assess the association between IgA-protease subtype and type of clinical infection.

## Results

A total of 230 isolates were included in the screening analysis. Of these, 177 isolates were from the collection of respiratory tract isolates; 43 isolates from January 2010, 52 isolates from January 2011 and 82 isolates from January 2012. In the 53 isolates comprising cohort 2, 42 isolates were from cases of invasive disease (26 from the bloodstream and 16 from cerebrospinal fluid (CSF)), 3 were from urogenital infections and the remaining 8 were encapsulated isolates from different origins. Of the invasive isolates, 33 were non-typeable (18 CSF and 15 bloodstream isolates). For a more detailed description of cohort 2, see Additional file 1: Table S1.

### Clinical descriptive features of patients in the respiratory tract cohort

The 177 isolates were collected from 177 unique patients. The descriptive features of patients from cohort 1 are presented in Table 1. The gender distribution was almost balanced, with a slight overrepresentation of women. The median age in the cohort was 32 years of age, 66 samples (37.7%) were from pre-school children (< 6 years of age). Approximately two thirds of isolates were collected in primary care facilities and one third in hospital care. Nineteen % of isolates were from cases of acute otitis media and 15% were from cases with lower respiratory tract infection. In 22% of patients, there was insufficient information in the referral text to identify the type of clinical infection.

**Table 1:** Cohort 1 comprising all respiratory tract isolates (*n* = 177). The distribution of clinical and laboratory characteristics per study year

Culture year	2010	2011	2012	Total
Number of isolates	43	52	82	177
*igaA1*, *n* (%)	31 (72%)	39 (75%)	51 (62%)	121 (68%)
*igaA1 alone*, *n* (%)	14 (33%)	29 (56%)	20 (24%)	63 (36%)
*igaA*2^a^, *n* (%)	12 (28%)	13 (25%)	30 (37%)	55 (31%)
*igaB1*^a^, *n* (%)	23 (53%)	15 (29%)	43 (52%)	81 (46%)
*igaB2*, *n* (%)	0	0	1 (1.2%)	1 (0.6%)
Age (years), mean (median)	24 (6)	32 (36)	29 (34)	29 (32)
Gender, % female	53%	67%	55%	58%
Primary care referrals, *n* (%)	31 (72%)	33 (63%)	55 (67%)	119 (67%)
Acute otitis media, *n* (%)	10 (24%)	8 (15%)	15 (18%)	33 (19%)
Sinusitis, *n* (%)	5 (12%)	5 (10%)	11 (13%)	21 (12%)
Upper respiratory tract infection, *n* (%)	17 (41%)	21 (40%)	18 (22%)	56 (32%)
Lower respiratory tract infection, *n* (%)	7 (17%)	8 (15%)	12 (15%)	27 (15%)
Unclear diagnosis from referral, *n* (%)	2 (5%)	10 (19%)	26 (32%)	38 (22%)

^a^Some isolates have both the *igaA* and *igaB* gene, resulting in a total percentage of > 100%

### The *iga* gene distribution in consecutive isolates from the respiratory tract

The PCR screening results performed for all 177 isolates of cohort 1 is presented in Table 1. The *igaA* gene PCR was positive in all but one isolate (176/177; 99%). The *igaA1* gene variant was found in 121/177 (68%) whereas the *igaA2* gene variant was found in 55/177 (31%) of isolates. Isolates also carrying the *igaB* gene were common throughout the study period, as 81/177 (46%) of isolates tested positive in the PCR screening. Only one of the isolates was *igaB2* positive. There was a year-to-year variation of the distribution, but no clear trend over time, with a lower proportion of *igaB*-positive isolates in 2011 compared with both 2010 and 2012, likely reflecting temporal variations in circulating strains.

### The *iga* gene distribution in cohort 2

The PCR screening results for all 53 isolates of cohort 2 is presented in Table 2. The *igaA* gene PCR was positive in all isolates. The capsule type-associated IgA-cleavage pattern from prior studies was mainly confirmed in this genotype analysis, as all included type b and d isolates had an *igaA1* genotype, and type c and e isolates had an *igaA2* genotype. Data on type f isolates have been conflicting as judged by prior studies, but in our study all four type f isolates included carried an *igaA2* genotype. In our two capsule type a isolates tested, the PCR for *igaB* screening was positive. Among the tested isolates in cohort 2, *igaB* was found in 11 isolates (21%), including 3 *igaB2*. Among all invasive isolates and invasive NTHi isolates *igaB* was found in 8/42 (19%) and 7/33 (21%) isolates, respectively. Two of the three included urogenital isolates had an *igaB* genotype. Information regarding isolation site and capsule type for cohort 2 is outlined in Additional file 1: Table S1. Information on the clinical descriptive features was, however, only available in a subset of the cohort.

**Table 2:** The distribution of laboratory characteristics per isolation site of the 53 isolates comprising cohort 2

Isolation site	Bloodstream	CSF	Other^b^	Total
Number of isolates	26	16	11	53
Encapsulated	8	1	6	15
Nonencapsulated	18	15	5	38
*igaA1*, *n* (%)	19 (73%)	11 (69%)	6 (55%)	36 (68%)
*igaA1* alone, *n* (%)	16 (62%)	8 (50%)	5 (45%)	26 (49%)
*igaA2*^a^, *n* (%)	7 (27%)	5 (31%)	5 (45%)	17 (32%)
*igaB1*^a^, *n* (%)	1 (4%)	4 (24%)	3 (27%)	8 (15%)
*igaB2*, *n* (%)	2 (8%)	1 (6%)	0	3 (6%)

^a^Some isolates have both the *igaA* and *igaB* gene, resulting in a total percentage of > 100%

^b^Available details on all isolates are given in Additional file 1: Table S1

### IgA1 cleavage pattern as compared to the diagnosed genotype

It is well established that a positive PCR screening does not necessarily correlate with protease activity. Therefore, we determined the IgA1 cleavage patterns by Western blot in a subset of 54 isolates. The isolates were chosen to represent all different genotypes, all years in cohort 1, in addition to all capsule types and isolation sites. Forty isolates were from cohort 1 (14 from 2010, 9 from 2011 and 17 from 2012), and 14 isolates from cohort 2 (representing all capsule types as well as isolation sites). Cleavage pattern A1 correlates with IgA-A1, cleavage pattern A2/B can be either IgA-A2 or IgA-B, and cleavage pattern A1 + A2/B indicates expression of two proteases simultaneously. Among 22 isolates tested with an *igaA1* genotype without *igaB*, 20 had indeed an A1 cleavage pattern. Among the 14 isolates analysed with an *igaA2* genotype without *igaB,* 13 had an A2/B cleavage pattern. In 11 out of 14 isolates tested with an *igaA1* + *igaB* genotype, the *igaB* gene was expressed; 9 had an A2/B cleavage pattern, while two had an A1 + A2/B cleavage pattern. Two isolates with an *igaB2* genotype were examined and only one of them had an A2/B cleavage pattern. Both analysed isolates with a combined *igaA2* and *igaB* genotype had an A2/B cleavage pattern. All tested encapsulated isolates had cleavage patterns corresponding to the genotype except for capsule type a, which had a A1 cleavage pattern, suggesting that the *igaB* gene is not expressed in *H. influenzae* type a. Taken together, in the majority of isolates the cleavage pattern phenotype corresponded to the genotype.

### Associations between the *iga* genotype and clinical predictors

Data of association and results from the regression analysis are presented in Tables 3 and 4, respectively. The *igaB* genotype was less common in invasive (bloodstream and CSF) isolates compared with isolates from other isolation sites. The difference was significant using Fischer’s test, 8/42 (19%) of invasive isolates compared with 84/188 (45%) of non-invasive isolates (*p* = 0.003), as well as for invasive non-typeable *H. influenzae* isolates alone, 7/33 (21%) compared with 83/182 (46%) of non-invasive isolates (*p* = 0.012). The negative association between *igaB* gene presence and bloodstream infection remained independently significant even when adjusting for covariates in a multinomial multivariate regression (Table 4). Younger age was associated with acute otitis media, whereas increasing age was associated with bloodstream infection in the same model. There was no significant association between *iga* genotype and type of respiratory tract infection (Table 3). A significant association between *igaA2* genotype and young age was also observed (Table 3).

**Table 3:** The *iga* protease gene variants among respiratory tract isolates with different properties

*iga* genotype	*igaA1* alone	*igaA2*	*igaB* gene present	*p*-value
Age (continuous, year), mean (median)	29.4 (21)	18.1 (4)	33.5 (40)	**< 0.001**
Age category (*n* = 175, 2 missing)				**0.042**
> 70 years, *n* (% within genotype)	5 (8%)	–	5 (6%)	
50–70 years, *n* (% within genotype)	14 (22%)	7 (13%)	20 (25%)	
20–50 years, *n* (% within genotype)	15 (24%)	14 (25%)	27 (34%)	
6–20 years, *n* (% within genotype)	5 (8%)	3 (5%)	6 (8%)	
< 6 years, *n* (% within genotype)	24 (38%)	31 (56%)	21 (27%)	
Gender, % female	56%	53%	62%	0.684
Primary care referrals, %	71%	65%	64%	0.966
Clinical diagnosis (*n* = 175, 2 missing)				0.172
AOM, *n* (% within genotype)	11 (17%)	13 (24%)	13 (16%)	
Sinusitis, *n* (% within genotype)	6 (10%)	6 (11%)	10 (13%)	
Upper respiratory tract infection, *n* (% within genotype)	26 (41%)	16 (29%)	24 (30%)	
Lower respiratory tract infection, *n* (% within genotype)	11 (17%)	3 (5%)	16 (20%)	
Unclear from referral, *n* (% within genotype)	9 (14%)	17 (31%)	16 (20%)	

The Kruskal-Wallis test for continuous variables and the chi-2 test for categorical variables were used. Significant *p*-values are indicated in bold

**Table 4:** Multinomial univariate and multivariate regressions with clinical diagnosis as the outcome (URTI was used as base category)

	Univariate analysis	Multivariate analysis
Covariate	Beta-coefficient (95% CI), *p*-value	Beta-coefficient (95% CI), *p*-value
Age	AOM vs URTI −0.087 (−0.13 - (−0.044)), **< 0.001** Sepsis vs URTI 0.038 (0.016–0.060), **0.001**	AOM vs URTI - 0.091 (− 0.14 – (− 0.047), **< 0.001** Sepsis vs URTI 0.041 (0.018–0.063), **< 0.001**
*igaB* vs all	Sepsis vs URTI − 1.69 (−3.00 – (− 0.38)), **0.012**	Sepsis vs URTI −1.86 (−3.22- (− 0.49)), **0.008**
*igaA2* vs all	no significant category	

The analysis was performed for isolates with *igaB* genotype vs all other isolates as well as those with *igaA2* genotype vs all other isolates. Only statistically significant associations are presented

## Discussion

In Sweden, the distribution of *iga* genotypes in *H. influenzae* was surprisingly consistent with the distribution described in the prior study performed on selected cohorts [11], as more than 40% of isolates from consecutive respiratory tract isolates were positive in the *igaB* gene screening. There was no significant association between the presence of the *igaB* gene and type of respiratory tract infection. We found a significant negative association between the presence of the *igaB* gene and invasive disease. This corresponds to the results in the study by Fernaays et al., where less than 20% invasive isolates carried the *igaB* gene compared with 30% of middle ear isolates and more than 40% of sputum isolates [11]. In the present material, 30% of CSF isolates possess the *igaB* allele, which is similar to middle ear isolates.

It has been suggested that IgA-A and IgA-B proteases have different roles in *H. influenzae* pathogenesis, and may be variably expressed during different phases of host colonization and invasion [13]. While IgA-A is implied in tissue colonization and invasion, IgA-B cleaves Lysosomal-associated membrane protein 1 (LAMP-1), which has been suggested to be important for bacterial persistence [14]. Bacterial persistence and colonization is likely less important than tissue invasion in cases of invasive disease. In addition, the expression of an additional immunogenic protein may hypothetically even be a negative factor in bloodstream survival of *H. influenzae* considering the limited serum survival capacity of the bacterium and potent humoral response elicited in most individuals [15]. One study has related the capacity to cause disease to differences in actual protease activity levels, where increased protease activity was suggested to be associated with increasing disease severity [16].

In prior studies, no association with specific pathogenesis or targeted population has been correlated with the *igaA2* variant. In this study, we found that *igaA2* was associated with young age, and was comparatively uncommon among individuals above 6 years of age. Furthermore, most *H. influenzae* type f isolates seem to have *igaA2*. Not much is known regarding the relative immunogenicity of the specific IgA protease variants. In children, a larger variety of strains have been suggested as necessary since antigenic type variation is important for colonization in children, in contrast to adults with chronic obstructive pulmonary disease (COPD) [17]. Thus, it could be speculated that IgA-A2 may have a smaller range of antigenic variation, a range potentially exhausted in early life for most patients. It has been suggested that strains with cleavage pattern A2/B has a smaller range of antigenic variation [8].

The distribution of *iga* genotypes in this study is similar to the distribution from a prior study from North America [9]. Non-typeable *H. influenzae* is often described as quite genetically diverse due to its natural competence, and subsequent horizontal gene transfer. This variation seems unlimited based on descriptions in the literature, but the core genome in *H. influenzae* has been successfully divided into a limited number of clades using Multi Locus Sequence Typing (MLST) or whole genome sequencing, with no clear geographical difference between clade distribution [18, 19]. Encapsulated isolates sort into specific clades while NTHi are scattered throughout the phylogenic tree, which still contains a finite number of clades. In the work by De Chiara and colleagues, all NTHi isolates with *igaB* were sorted into two distinct clades [19]. Our results, in comparison with results from the North American study, may suggest that the distribution of genetic clades of respiratory tract NTHi might be relatively consistent across geographical regions.

The main strengths of the study include the tested population-based isolate collection, including non-selected consecutive respiratory tract isolates from three different years with associated clinical information as well as a collection of encapsulated and non-encapsulated isolates of varying origin. Another strength is the statistical comparisons included. Limitations of the study include its modest size and the differences in origin and isolation years between cohort 1 and cohort 2. This is also the reason why the cohorts have been treated separately.

## Conclusions

To summarize, our data demonstrate that the *igaB* genotype is frequently found in respiratory tract isolates from patients in the County of Skåne, Sweden with respiratory tract infections, a finding consistent over a 3-year period of observations. We also demonstrate that the *igaB* genotype was less frequently seen in invasive isolates compared to isolates from the respiratory tract. Finally, the *igaA2* genotype seems to be particularly relevant for respiratory tract infections in children (< 6 years).

## Methods

### Study setting and isolate selection

This study was performed in Malmö, Sweden, at a laboratory of clinical microbiology serving an area with a population of approximately 500,000 patients. It was the only microbiology laboratory in the region. Two cohorts were investigated. In the first cohort, all *H. influenzae* isolated from respiratory tract samples in January 2010, 2011 and 2012 (all tested as non-typeable) were selected for further testing. This strategy was chosen to allow for temporal variations in the distribution. The second cohort comprised of a collection of well-characterized invasive and encapsulated isolates from cases with different clinical infections. The cohort includes Swedish isolates from 1997 to 2010 and international isolates representing all capsule types. Details on isolation sites and origins of isolates in cohort 2 are given in Additional file 1: Table S1.

### Culture conditions and DNA preparation

All isolates were grown on chocolate agar plates overnight (18 h) in a humid atmosphere at 37 °C and 5% CO_2_ before any experiments were conducted. All isolates had been confirmed as *H. influenzae* using standard microbiology techniques or MALDI-TOF (Matrix Assisted Laser Desorption and Ionization - Time-of-flight mass spectrometry). DNA was prepared by adding a few colonies of bacteria to distilled water. After heating at 98 °C for 10 min, each sample was centrifuged (16,000 x *g*) followed by collection of the supernatant.

### PCR screening

**Fig. 1:**
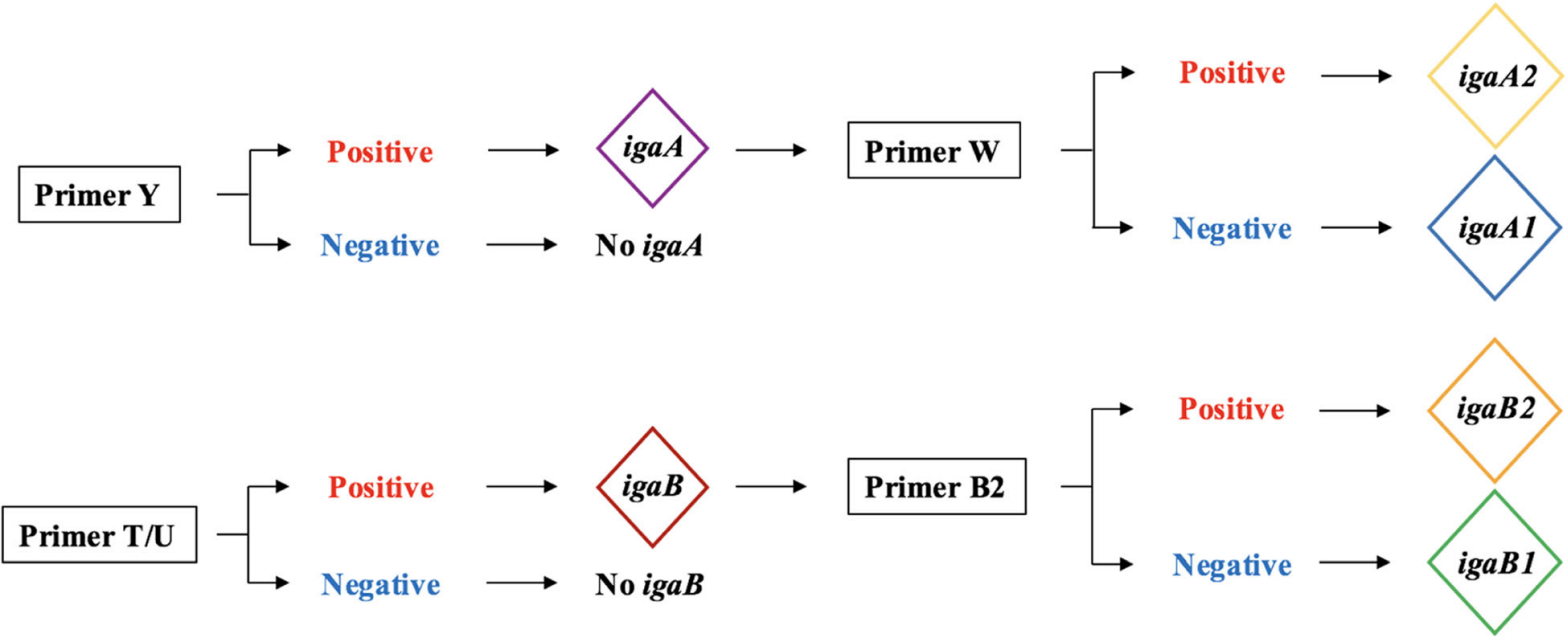
Sequential genotyping used to identify the different *iga* alleles

All isolates were screened using four different PCR primer pairs as described in prior studies [12], Y (identifying the *igaA* gene), W (identifying the *igaA2* variant of the *igaA* gene) as well as T and U (identifying the *igaB* gene). Isolates positive in the *igaB* screening were subjected to a fifth PCR, identifying the *igaB2* variant. The genotyping algorithm is visualized in Fig 1. Conditions and primer sequences are outlined in Additional file 2: Table S2. DNA was separated using a 1xTAE 1% agarose gel with 0.625 mg/mL ethidium bromide. Bands were visualized with UV light using a BioRad molecular Imager (Hercules, CA).

### IgA1 cleavage analysis by Western blot

A subset of 54 isolates, representing all IgA protease gene variants, was chosen for confirmation of IgA1 cleavage by Western blot. The strains were grown in brain-heart-infusion (BHI) broth supplemented with 10 μg/ml NAD and hemin at 37 °C 5% CO_2_ overnight. The bacteria were spun down and 20 μl of supernatant was incubated with 3 μl of 1 mg/ml human IgA1 (Calbiochem, Solna, Sweden) at 37 °C overnight. The proteins were separated by SDS-PAGE and transferred to nitrocellulose membranes. The membranes were incubated with polyclonal rabbit anti-human IgA antibodies at dilution 1:1000 overnight at 4 °C (A0262, Dako; Glostrup Denmark) followed by polyclonal HRP (horseradish peroxidase)-conjugated swine anti-rabbit IgG (dilution 1:1000) (P0217, Dako; Glostrup, Denmark) 2 h at room temperature and developed.

### Information on clinical infection

In the cohort of respiratory tract isolates, the information regarding type of clinical infection was gathered from the referral text. The disease types were lower respiratory tract infection, acute otitis media, sinusitis or unspecfic upper respiratory tract infection. We also collected data on patient age, gender and whether the culture had been taken in primary care or at a hospital facility. In the second cohort, information on capsule type and isolation site (blood, CSF or other) was available, and in a subset of cases also age and gender.

### Statistical analysis

Data analysis was performed using STATA, version14 (College Station, TX). The presence or absence of *igaB* between invasive and non-invasive isolates was assessed using a two-sided Fischer’s exact test. Comparison of clinical traits among isolates with different genotypes was performed using the Kruskal-Wallis test for continuous variables and the chi-2 test for categorical variables. To assess the relevance of confounders in the associations between IgA1 protease genotypes and clinical diagnoses, multinomial multivariate logistic regression models using categorical clinical diagnosis as outcome were fitted using the purposeful selection algorithm [20]. Briefly, the main predictor (IgA protease genotype, *igaA2* vs all and *igaB* vs all, respectively) and all covariates that in a univariate analysis had a *p*-value of < 0.2 were included in a crude model. The least significant covariate was then stepwise removed until only significant covariates or covariates that substantially affected the odds ratio of a remaining covariate were retained in the final model.

## Additional files

Additional file 1: **Table S1.** A more detailed description of isolates comprising cohort 2. (DOCX 20 kb)

Additional file 2: **Table S2.** Primers and PCR conditions. (DOCX 18 kb)

## Abbreviations

BHI: Brain-heart-infusion
COPD: Chronic obstructive pulmonary disease
CSF: Cerebrospinal fluid
DNA: Deoxyribonucleic acid
HRP: Horseradish peroxidase
IgA: Immunoglobulin A
IgG: Immunoglobulin G
LAMP-1: Lysosomal-associated membrane protein 1
MALDI-TOF: Matrix assisted laser desorption and ionization - Time-of-flight mass spectrometry
MLST: Multi locus sequence typing
NTHi: Non-typeable *Haemophilus influenzae*
PCR: Polymerase chain reaction
