# Supplementary material for: Differential distribution of IgA-protease genotypes in mucosal and invasive isolates of Haemophilus influenzae in Sweden
Source: BMC Infect Dis. 2018 Nov 22;18:592. doi: 10.1186/s12879-018-3464-3 (PMC6249890; doi:10.1186/s12879-018-3464-3)
Supplement: Supplementary file 1 — Table S1. A more detailed description of isolates comprising cohort 2. (DOCX 20 kb) [file 12879_2018_3464_MOESM1_ESM.docx]

**Additional Table 1.** Description of isolates included in cohort 2.

| **Strain** | **Year** | **Capsule type** | **Origin** | **Isolation site** |
| --- | --- | --- | --- | --- |
| HK390 | 1941 | Hia | USA | Resp. tract |
| HK391 | 1942 | Hia | - | CSF |
| HK395 | 1954 | Hib | - | not known |
| HK396 | 1942 | Hic | USA | sputum |
| HK635 | 1980 | Hic | Papua New Guinea | Resp. tract |
| HK644 | 1979 | Hid | Papua New Guinea | not known |
| HK653 | 1979 | Hie | Papua New Guinea | not known |
| HK880 | 1987 | NTHi | Brazil | Conjunctivae |
| HK865 | 1988 | NTHi | Brazil | Conjunctivae |
| HK1105 | 1993 | NTHi | France | Blood |
| HK1110 | 1993 | NTHi | France | Blood |
| M56 | 1997 | NTHi | Sweden | CSF |
| KR186 | 2000 | NTHi | Sweden | CSF |
| M44 | 2003 | NTHi | Sweden | CSF |
| KR198 | 2004 | NTHi | Sweden | CSF |
| L68 | 2004 | NTHi | Sweden | CSF |
| M42 | 2004 | NTHi | Sweden | CSF |
| KR194 | 2004 | Hib | Sweden | Blood |
| L2 | 2004 | Hib | Sweden | Blood |
| L38 | 2004 | NTHi | Sweden | Blood |
| L40 | 2004 | Hib | Sweden | Blood |
| G9 | 2005 | NTHi | Sweden | CSF |
| KR207 | 2005 | NTHi | Sweden | CSF |
| L6 | 2005 | NTHi | Sweden | CSF |
| M64 | 2005 | NTHi | Sweden | blood |
| L10 | 2006 | NTHi | Sweden | CSF |
| L14 | 2006 | NTHi | Sweden | CSF |
| M28 | 2006 | NTHi | Sweden | CSF |
| L49 | 2006 | NTHi | Sweden | blood |
| M65 | 2006 | Hif | Sweden | blood |
| KR222 | 2007 | NTHi | Sweden | CSF |
| L55 | 2007 | NTHi | Sweden | blood |
| L56 | 2007 | NTHi | Sweden | blood |
| M25 | 2007 | NTHi | Sweden | blood |
| M18 | 2007 | NTHi | Sweden | blood |
| G35 | 2008 | NTHi | Sweden | CSF |
| KR235 | 2008 | NTHi | Sweden | CSF |
| KR225 | 2008 | NTHi | Sweden | blood |
| KR227 | 2008 | NTHi | Sweden | blood |
| KR232 | 2008 | NTHi | Sweden | blood |
| M10 | 2008 | Hif | Sweden | blood |
| M11 | 2008 | NTHi | Sweden | blood |
| M12 | 2008 | Hif | Sweden | blood |
| KR223 | 2008 | NTHi | Sweden | blood |
| G27 | 2009 | NTHi | Sweden | blood |
| L26 | 2009 | NTHi | Sweden | blood |
| M1 | 2009 | Hif | Sweden | blood |
| M5 | 2009 | NTHi | Sweden | blood |
| G40 | 2009 | NTHi | Sweden | blood |
| AG17698 | 2012 | NTHi | Sweden | cervix |
| AG4948 | 2012 | NTHi | Sweden | cervix |
| AG 2690 | 2013 | NTHi | Sweden | cervix |
| ST45 Kansas | 2013 | Hib | USA | blood |
