# Supplementary material for: Differential distribution of IgA-protease genotypes in mucosal and invasive isolates of Haemophilus influenzae in Sweden
Source: BMC Infect Dis. 2018 Nov 22;18:592. doi: 10.1186/s12879-018-3464-3 (PMC6249890; doi:10.1186/s12879-018-3464-3)
Supplement: Supplementary file 2 — Table S2. Primers and PCR conditions. (DOCX 18 kb) [file 12879_2018_3464_MOESM2_ESM.docx]

**Additional Table 2**. Primers and PCR conditions.

| **Target gene** | **Primer** | **Annealing temp.** | **Amplicon size** | **Primer name** | **Sequence** |
| --- | --- | --- | --- | --- | --- |
| *igaA* | Y | 49 °C | ≈800 bp | Y1 | GCAAAAGCACAATTTGTTGCA |
|  |  |  |  | Y2 | TTATAACGTTAATTCAAACAGGCTT |
| *igaA2* | W | 42 °C | 548 bp | W1 | CAATATATTGTAAGCGTA |
|  |  |  |  | W2 | CTAATACGCCGTAGTTGG |
| *igaB* | T | 45 °C | 1004 bp | T1 | TTCTTCGCCAAAGAAACCGC |
|  |  |  |  | T2 | ATCTATAAAAAAGAATTTGC |
| *igaB* | U | 52 °C | 139 bp | U1 | AGGCAATGTCTTGTTATCAGGTCG |
|  |  |  |  | U2 | TTTGAATGTGCGGTTTATCCAGTC |
| *igaB2* | IgAB2 | 46 °C | ≈225 bp | IgAB2for | AATTTTTCTTTCAATGT |
|  |  |  |  | IgAB2rev | TCACTAGTGCAGCCTC |
